# Choosing an Animal Model for the Study of Functional Dyspepsia

**DOI:** 10.1155/2018/1531958

**Published:** 2018-02-12

**Authors:** Yang Ye, Xue-Rui Wang, Yang Zheng, Jing-Wen Yang, Na-Na Yang, Guang-Xia Shi, Cun-Zhi Liu

**Affiliations:** ^1^Acupuncture and Moxibustion Department, Beijing Hospital of Traditional Chinese Medicine Affiliated to Capital Medical University, Beijing Key Laboratory of Acupuncture Neuromodulation, Beijing, China; ^2^Beijing University of Chinese Medicine, Beijing, China

## Abstract

Functional dyspepsia (FD) is a common functional gastrointestinal disorder with pain or discomfort in the upper abdomen as the main characteristic. The prevalence of FD worldwide varies between 5% and 11%. This condition adversely affects attendance and productivity in the workplace. Emerging evidence is beginning to unravel the pathophysiologies of FD, and new data on treatment are helping to guide evidence-based practice. In order to better understand the pathophysiologies of FD and explore better treatment options, various kinds of animal models of FD have been developed. However, it is unclear which of these models most closely mimic the human disease. This review provides a comprehensive overview of the currently available animal models of FD in relationship to the clinical features of the disease. The rationales, methods, merits, and disadvantages for modelling specific symptoms of FD are discussed in detail.

## 1. Introduction

Functional dyspepsia (FD) is a highly prevalent gastrointestinal disorder that is clinically characterized by diverse symptomatology including a sensation of pain or burning in the epigastrium, postprandial fullness, early satiety, bloating, and nausea [1]. The global prevalence of uninvestigated dyspepsia in adults is 20.8%, but this figure varies depending on the geographical location and the definition of disease [2]. Although the pathological mechanisms underlying FD have not yet been fully elucidated, existing research indicates that the etiology of FD is multifactorial. Delayed gastric emptying, impaired gastric fundus accommodation, visceral hypersensitivity, and various psychosocial factors are considered to be the major pathophysiologic disturbances of FD [3].

Animal models have been extensively used to determine pathological mechanisms and to develop new therapies for human diseases including FD. Many attempts have been performed to model various aspects of FD. Results from these animal studies have benefited our understanding of the pathophysiology of this disease and helped to identify potential therapies [4]. Despite developments in our understanding of FD, scientists around the world have not yet developed a specific treatment or preventive drug for this disorder [5]. Therefore, FD animal models will continue to have a critical role in drug screening and therapy development, which could eventually lead to potential therapeutic strategies for FD patients. Various types of FD animal models are employed in the current preclinical studies [4]. A common question about the FD animal models is which of these models most closely simulate the clinical and pathologic features of the human disease?

In this review article, we will summarize progress that has been made in modelling FD in experimental animals from the perspective of the pathophysiological features and clinical symptoms. The animals used for in vivo FD studies have included rats, mice, and dogs. But here, we will primarily focus on rat models because rats are the most commonly used animals for modelling FD (Table 1). Understanding the fundamental methodology, strengths, and weaknesses of the various kinds of modelling methods will help in the choice of the most suitable model for a particular purpose and question of interest. Furthermore, we will propose developmental directions of FD animal model for future studies.

## 2. Pathophysiological Features of FD

### 2.1. Delayed Gastric Emptying

Delayed gastric emptying is considered to be a pathophysiological feature of FD that is closely related to dyspepsia symptoms [41]. A study showed that the prevalence of delayed emptying in FD patients ranges between 20 and 35% [42]. When food moves to the small intestine FD symptoms including fullness, bloating, and belching develop [43]. Many prokinetic agents have been used in the treatment of FD, such as cisapride, domperidone, and itopride [44].

### 2.2. Impaired Gastric Fundic Accommodation

Gastric accommodation is mediated by the activation of noncholinergic nerves in the gastric wall that result in the production and diffusion of nitric oxide to gastric smooth muscles [45]. Impaired gastric fundic accommodation, a vagal reflex, refers to slow gastric emptying and the failure of the gastric fundus to reflex after a meal [46]. Impaired accommodation is a frequently encountered pathophysiological abnormality in FD [47]. Research has shown that 40% of dyspeptic patients have impaired accommodation, and this is associated with early satiety and weight loss [48].

### 2.3. Visceral Hypersensitivity

Visceral hypersensitivity has been considered to play an important role in the generation of FD symptoms [49]. Data show that 30% of patients with functional dyspepsia have evidence of hypersensitivity to gastric distention [50]. In addition, the duodenum is recognized as a site involved in symptom generation in FD through increased sensitivity to acid and lipids [51]. Visceral hypersensitivity may result from alterations in the peripheral or central nervous system and the etiology is complex [52]. Stimuli including hollow organ distension, inflammation, traction on the mesentery, and ischemia may lead to visceral hypersensitivity under the pathological circumstances [53]. Visceral hypersensitivity to distention in FD is associated with symptoms of early satiety, abdominal pain, postprandial pain, excessive belching, nausea, and unexplained weight loss [54].

### 2.4. Psychological Distress

Psychological distress including anxiety and depression is related to FD and may precede the onset of FD in some people [55]. The relationship of FD with anxiety and depression is complex and may be associated with processing of negative stimulus in the central nervous system and brain-gut axis [56]. The detection rates of depression and anxiety symptoms in patients with functional dyspepsia are 34.36% and 25.55%, respectively [57]. Because of the potential role of psychological distress in FD, antidepressants have been recommended as a therapy [3].

## 3. Drug Administration Animal Models

### 3.1. Clonidine Injection

*Rationale.* Sympathetic and parasympathetic control of gastric motility is a classic example of norepinephrine and acetylcholine triggering opposing actions [58, 59]. It is well known that both norepinephrine and acetylcholine are important regulators of gastrointestinal motility, and inhibition to acetylcholine degradation has been reported to enhance gastric motility [6]. As an *α*_2_-adrenoceptor agonist, clonidine is used to suppress the release of acetylcholine from cholinergic neurons. This could ultimately lead to hypomotility, delayed gastric emptying, and intestinal transit [60, 61].

*Methods. *In the clonidine-induced motility dysfunction model, clonidine is subcutaneously administered to animals [7].

*Features. *Clonidine injection mainly leads to motility dysfunction including hypomotility and delayed gastric emptying by inhibiting acetylcholine activity [6–8]. There are no reports to suggest that this model could be used to induce other pathological features of FD, such as impaired fundic accommodation or visceral hypersensitivity. In addition, the motility dysfunction caused by clonidine is temporary in animals, so gastrointestinal motility should be evaluated shortly after the clonidine administration. Therefore, this is a transitory FD animal model which specifically focuses on the gastrointestinal motility dysfunction.

### 3.2. Atropine Injection

*Rationale. *The effects of acetylcholine on gastrointestinal contractility are mainly regulated by muscarinic acetylcholine receptors [62]. So the activity of muscarinic acetylcholine receptors is directly tied to the gastrointestinal motility [63]. Atropine, a muscarinic acetylcholine receptor antagonist, could be used to induce delay in gastrointestinal transit [10].

*Methods. *Different methods exist to establish an atropine-induced FD animal model. Atropine can be injected subcutaneously or intraperitoneally to make a delayed gastrointestinal transit model [10, 11].

*Features. *This is another motility dysfunction model that exerts its effect by directly acting on the cholinergic system. It also persists for a short time and only induces delayed gastrointestinal transit.

### 3.3. Dopamine Injection

*Rationale.* Dopamine inhibits acetylcholine release and gastric motility and it has been proposed as a possible neurotransmitter in gastric relaxation [64, 65]. The effect of dopamine has been thought to be mediated through the dopamine-2 receptor and dopamine-3 receptor [65]. Therefore, dopamine could be used to induce delayed gastric emptying in FD animal studies.

*Methods. *In this model, dopamine is intraperitoneally administered to animals [9, 10].

*Features.* This is also a transient delayed gastric emptying FD model. As dopamine is degraded, the delayed gastric emptying will return to normal again.

### 3.4. Apomorphine Injection

*Rationale. *Dopamine delays gastric emptying, and inhibiting the effect of dopamine is achieved by activating dopamine receptors [65]. As a dopamine receptor agonist, apomorphine inhibits gastrointestinal motor function [12, 66].

*Methods. *Animals are subcutaneously injected with apomorphine to induce delayed gastric emptying [12, 13].

*Features. *This model is similar to the dopamine-induced delayed gastric emptying model. It could be used as a FD model when gastric motility needs to be evaluated.

### 3.5. 5-Hydroxytryptamine Injection

*Rationale. *5-Hydroxytryptamine (5-HT) plays an important role in the regulation of gastrointestinal motility [67]. Gastrointestinal contraction amplitude is increased by 5-HT primarily via a cholinergic pathway. Furthermore, 5HT_3_ receptors and 5HT_1_-like and/or 5HT_2C_ receptors are responsible for 5HT-induced gastrointestinal contractions [67].

*Methods.* In this model, 5-HT is intraperitoneally injected into animals [10].

*Features. *5-HT is used to induce reductions in gastric emptying and small intestinal motility. It is a short-term FD model which only concentrates on impaired gastrointestinal motility.

### 3.6. 5-HT3 Receptor Agonist Injection

*Rationale. *One of the most well-established physiological roles of the 5-HT_3_ receptor is to regulate gastrointestinal motility [68]. 5HT-induced inhibition of gastrointestinal dynamics might be partially achieved via 5HT_3_ receptors [67]. 5-HT_3_ receptor agonists suppress gastric emptying and gastrointestinal motility [10].

*Methods.* A 5-HT_3_ receptor agonist is intraperitoneally injected into animals to induce a reduction in gastric emptying and small intestinal motility [10].

*Features. *This model is similar to the 5-HT-induced delayed gastric emptying model. The motility of the small intestine is reduced by 5-HT_3_ receptor agonist injection [10]. In another study, m-chlorophenylbiguanide (m-CPBG) was employed to induce a FD animal model as a selective 5-HT_3_ receptor agonist [9].

### 3.7. Corticotropin-Releasing Factor Injection

*Rationale. *Acute stress induces a delay in gastric emptying through a peripheral sympathetic pathway [69]. Corticotropin-releasing factor (CRF), a pituitary hormone secreted in response to stress, is closely related to stress-induced abnormal colonic responses such as stimulation of motility, transit, defecation, and occurrence of diarrhea [70].

*Methods.* CRF is intravenously injected into animals in this model [14].

*Features.* Delayed gastric emptying is induced by CRF injection and this effect is temporary. CRF has been reported to be involved in visceral hypersensitivity, but this role of CRF has not been investigated in FD animal studies [70].

### 3.8. Cisplatin Injection

*Rationale. *Cisplatin, an effective chemotherapeutic agent that has been widely used in the treatment of various cancers, causes severe side effects on gastrointestinal function such as a delay in gastric emptying as well as nausea and vomiting [11]. Delayed gastrointestinal transit induced by cisplatin could be used to mimic the impaired gastric motility of FD.

*Methods.* Cisplatin is intraperitoneally injected to animals to induce inhibition of gastric emptying [11, 15].

*Features.* Cisplatin injection induces delayed gastric emptying, which could be used to mimic the gastrointestinal motility dysfunction of FD, but cisplatin also causes many other serious side effects such as kidney and liver damage, which is a drawback of this method [71, 72].

### 3.9. Soybean Oil Administration

*Rationale. *Soybean oil, a common dietary fat, is mostly used for frying and baking. In biomedical science, soybean oil is commonly used to investigate biological reactions to dietary fat [73]. It is known that fat can delay gastric emptying [74, 75]. Soybean oil has been used to induce delayed gastric emptying model [14, 76].

*Methods.* After the deprivation of food and water, soybean oil is orally administrated to animals [14].

*Features. *This model uses soybean oil to inhibit gastric emptying in a dose-dependent manner [14].

## 4. Neonatal Intervention Animal Models

### 4.1. Neonatal Gastric Irritation

*Rationale. *This FD model refers to the neonatal administration of a mild irritant, which results in transient superficial sloughing of the gastric epithelium [16]. Iodoacetamide has been reported to cause mild gastritis, along with impairments of gastric sensory and motor function [77, 78]. The neonatal period is a time of known neuronal vulnerability to long-term plasticity [79, 80]. Therefore, gastric irritation with iodoacetamide in the neonatal period leads to gastric hypersensitivity and motor dysfunction that persist into adulthood without significant morphologic and histologic changes in the stomach [16–19].

*Methods. *In this model, animal pups are given 0.1% iodoacetamide in a sucrose solution by oral gavage daily for six consecutive days. After the gastric irritation, animals are then fed normally until adulthood [16].

*Features.* Neonatal iodoacetamide treatment early in life induces gastric hypersensitivity, motor dysfunction, and impaired accommodation in adulthood. In addition, gastric irritation in the neonatal period also leads to a long-lasting increase in depression-like and anxiety-like behaviors [20]. Overall, neonatal gastric irritation is a classical and comprehensive method to induce a FD animal model. A clear understanding of the mechanisms responsible for the pathophysiology of this model need to be elucidated in future studies.

### 4.2. Neonatal Colon Inflammation

*Rationale.* Trinitrobenzene sulfonic acid (TNBS) induces chronic colonic inflammation in rodent animals [81]. In this pathological process, prostanoid concentrations are increased causing macroscopic and microscopic gastrointestinal lesions [82, 83]. In addition, intraluminal administration of TNBS also induces FD-like gastric hypersensitivity and anxiety behaviors [22, 23]. Early life inflammation leads to a high risk for the development of FD in adulthood [84, 85].

*Methods. *Animal pups receive TNBS acid in 10% ethanol in saline through a catheter inserted 2 cm into the distal colon on postnatal day 10 without anesthesia. The animals are kept in a head-down position for about 2 minutes during the administration of TNBS and the anus is held closed for 1 minute to prevent leakage of the TNBS solution. Six to eight weeks later, the now-adult animals are used as an FD model [21, 22].

*Features. *TNBS-induced colon inflammation is a classical method to induce a colitis animal model. This model presents multiple pathological characteristics of FD including gastric hypersensitivity, delayed gastric emptying, and depression-like behaviors [21–24]. Therefore, it is another comprehensive model of FD. Due to the toxicity of TNBS, the mortality rate of neonatal colon inflammation was 4.1% [22].

### 4.3. Neonatal Maternal Separation

*Rationale. *Adverse physiological or psychological experiences in early life are associated with the development of FD symptoms [84, 86]. Childhood trauma might lead to a more sensitive response of the hypothalamus-pituitary-adrenal (HPA) axis, motor dysfunction of the gastrointestinal tract, and dyspeptic symptoms in adult FD patients [25]. Neonatal maternal separation is a well-established experimental model of early life stress which has been used in many studies [87, 88].

*Methods. *Animal pups are removed from their mother's cage and placed in an individual cage for three consecutive hours daily. This process lasts for 12 or 19 days [25, 26]. The pups are returned to their mother cage after maternal separation. On postnatal day 22, all pups are weaned. These animals are used in FD models when they reach adulthood. To avoid hormonal cycle-induced variations, only male pups are used.

*Features.* Exposure of animals to neonatal maternal separation leads to structural changes in gastric enteric glial cells and a delay in gastric emptying. The effect of inducing FD is limited when neonatal maternal separation is used independently. Therefore, it is better to combine neonatal maternal separation with another method to establish a good FD animal model [25, 26].

## 5. Stress Stimulation Animal Models

### 5.1. Restraint Stress

*Rationale. *Psychological and physiological stresses such as restraint stress have been shown to inhibit antral motility and gastric emptying in animals [89–91]. Delayed gastric emptying is related to the stress-induced sympathetic activation, which is accompanied by increased stress hormones and active ghrelin [26]. Furthermore, stress hormones and active ghrelin play key roles in mediating gastric motility [26, 92].

*Methods. *Animals are restrained in cylindrical, well-ventilated, and stainless steel tubes for 60 or 90 min once a day for seven days [26–28].

*Features. *In this model, physiologic and psychological stresses are used to induce feeding inhibition and delayed gastric emptying. Chronic stress is established in this animal model because the stress status lasts for seven days.

### 5.2. Water-Immersion Restraint Stress

*Rationale. *Mental and physical stress exert an inhibitory effect on the gastrointestinal tract via various mediators including catecholamine, CRF, and ghrelin [93, 94]. Acute stressors may have profound effects on intestinal epithelial physiology, stimulating ion secretion, and reducing barrier function [95]. The model of water-immersion restraint stress produces both physical and psychological stresses [29].

*Methods.* The total body of the animal from head to lower hind limbs is tightly placed in cages, and the entire body except the head is immersed vertically to the level of the xiphoid process in a water bath for several hours. The stress session is performed only once [25, 29].

*Features. *This stress model is reported to increase plasma adrenocorticotrophic hormone (ACTH) and cortisol levels [29]. It also induces delay in gastric emptying [25]. However, this is an acute stress model instead of a chronic stress model, which may differ from human pathophysiology.

### 5.3. Tail Clamping

*Rationale. *Stress is found to contribute to gastrointestinal motility disorders [96]. Tail clamping angers animals and causes fighting, which may lead to anxiety-like behaviors and delayed gastric emptying.

*Methods.* Animals' tails are clamped with sponge forceps distally to anger them and cause fighting. This is performed for 30 minutes at a time, but without injury. This procedure is repeated four times daily for seven continuous days [30–32].

*Features. *Animals appear dispirited, irritable, nervous, and anxious and lose their appetite after tail clamping. The gastric myoelectrical main frequency, power, and the percentage of slow wave duality of the animals decrease significantly. Tail clamping also induces delay in gastric emptying and an increase in serum nitric oxide [32]. It is another chronic stress model which combines mental and physical stresses.

### 5.4. Chronic Mild Unpredictable Stimulation

*Rationale. *Chronic mild unpredictable stimulation (CMUS) is a classical method to induce depression in animals [97]. Patients with FD have been reported to score highly for anxiety and depressive symptoms [57]. The depression-like behaviors due to CMUS are similar to symptoms of some FD patients.

*Methods. *FD is established through CMUS, which included bondage, swim-induced fatigue, electrical stimulation, fasting, and concussion. The stimulation is performed every day for three continuous weeks [33].

*Features. *CMUS is a common experimental depression model. This model could be used to focus on depression in FD patients. After CMUS, the weight of animals decreases and the numbers of crossings, cleanings, and stand-up times decrease.

## 6. Other Intervention Animal Models

### 6.1. Gastric Distension

*Rationale.* Visceral hyperalgesia plays an important role in the pathophysiology of FD and may partially result from sensitization of primary afferent fibers innervating the gastrointestinal tract [98]. The visceromotor response to gastric distension could be used to study visceral hyperalgesia in conscious animals. Abdominal muscle contractions in response to balloon gastric distention are regarded as an indicator of visceral pain.

*Methods.* A flexible latex balloon catheter is surgically placed into the stomach though a small incision. A force transducer is then sutured to the abdominal external oblique muscle to measure the number of abdominal contractions as an indicator of visceral pain sensation [34].

*Features. *Gastric distension-induced visceromotor response is a special FD model which replicates visceral hyperalgesia for FD. The limitation of this model is that it does not reproduce other aspects of FD, such as delayed gastric emptying, impaired gastric fundic accommodation, and depression-like behaviors.

### 6.2. Laparotomy

*Rationale. *Laparotomy followed by intestinal manipulation has been used as a classical method to induce postoperative ileus animal model [99]. After laparotomy and intestinal manipulation, spinal afferents and the motor nucleus of the vagus nerve are activated. They activate many inhibitory pathways involving adrenergic neurons and nitrergic and vipergic neurons, which then reduce the motility of gastrointestinal tract. After the early neurogenic phase, local inflammation becomes the leading cause of continuous gastrointestinal hypomotility [100].

*Methods.* After a rat is anesthetized, a laparotomy is performed using a three-centimeter midline incision. The small intestine and caecum are gently pulled out of the abdominal cavity and the small intestine is gently manipulated with the fingers for ten minutes. After manipulation, the small intestine and caecum are replaced and the surgical wound is sutured [35].

*Features. *Laparotomy can be used to establish animal model of delayed gastrointestinal transit. It is easy to perform and the results are reliable. However, this model only mimics impaired gastrointestinal motility of FD.

### 6.3. Duodenal Acidification

*Rationale.* Hypersensitivity in gastric perception is considered to be one cause of FD [3]. Clinical studies have shown the involvement of duodenal acidification-induced gastric hypersensitivity in the pathogenesis of FD [101, 102].

*Methods.* 0.01 mol/L hydrochloric acid (HCI) is infused into the proximal duodenum at a rate of 0.1 ml/min [36].

*Features. *Duodenal infusion of HCl enhances the gastric nociceptive response in conscious animals. This model is able to accurately mimic the clinical findings of gastric hypersensitivity induced by duodenal acidification.

### 6.4. Chronic High-Fat Feeding

*Rationale. *High-fat feeding is relevant to FD and bile acids affect gastrointestinal motility [103, 104]. Increased levels of circulating bile acids induced by chronic high-fat feeding upregulate neuronal nitric oxide synthase (nNOS) and bile acid receptor 1 (TGR5) expression in the gastric myenteric plexus, resulting in enhanced noncholinergic relaxation and delayed gastric emptying [37].

*Methods.* Animals are fed a high-fat diet for two consecutive weeks. The high-fat diet includes 58% kcal fat [37].

*Features. *High-fat feeding for two weeks leads to delay in gastric emptying. This model mimics the clinical situation of FD from a dietary perspective. It is a useful tool to investigate effective treatments for FD induced by chronic high-fat feeding.

## 7. Special Species Animal Models

### 7.1. Flinders Sensitive Line Rats

*Rationale. *Flinders Sensitive Line (FSL) rats, selectively bred for hypersensitivity to cholinergic stimuli, were originally proposed as a genetic animal model of depression [105]. Like some depressed humans, FSL rats show hypersensitivity to cholinergic agonists, are less active, exhibit higher rapid eye movement sleep, and respond to antidepressants [106, 107]. Anxiety and depression are associated with FD and may precede the onset of the disorder in some people [55]. As the FSL rats show some behavioral similarities to FD patients, this species could be used in FD models.

*Features. *FSL rats have disturbed gastric motility, reflected as both an increased gastric accommodation rate and gastric volume during gastric distension, which then leads to a delay in gastric emptying [38]. Although FSL rats show some features similar to some FD patients, the increase in gastric accommodation in FSL rats is contrary to the reduced accommodation often seen in FD patients. FSL rats may be useful to study certain aspects of FD, such as depression and delayed gastric emptying.

### 7.2. Wistar Kyoto Rats

*Rationale.* Wistar Kyoto (WKY) rats, a high-anxiety strain considered to be hyperresponsive to stress, are also a recognized animal model of impaired gastric accommodation [108, 109]. The WKY rats exhibit some of the pathophysiological characteristics of some FD patients, such as impaired gastric accommodation and anxiety.

*Features. *During gastric distension, WKY rats have a lower intragastric volume, indicating impaired gastric accommodation. The impaired gastric accommodation seen in WKY is associated with an increased cholinergic activity of the gastric vagal nerves [109]. In WKY rats, impaired accommodation of smooth muscles might not be a widespread phenomenon along the gastrointestinal tract but rather a local disturbance [39]. WKY rats may be useful to study certain FD patients who have both impaired gastric accommodation and anxiety.

### 7.3. BioBreeding Diabetes-Prone Rats

*Rationale. *Normoglycemic BioBreeding diabetes-prone (BB-DP) rats display both altered fundic motor control and impaired gastric accommodation, which is at least partially caused by a loss in nitrergic nervous function. Normoglycemic BB-DP rats provide a spontaneous model for inflammation-induced impaired gastric accommodation [40].

*Features. *This impaired accommodation is associated with low-grade inflammation and loss of neuronal isoform of nitric oxide synthase proteins which develop over time in this model [40]. The normoglycemic BB-DP rats may help us to understand the pathophysiological mechanisms in FD patients with inflammation-induced impaired gastric accommodation and develop new medications for these people.

## 8. Animal Models Multiple Features

### 8.1. Neonatal Colon Inflammation Followed by Adult Gastric Irritation

*Rationale.* Neonatal colon inflammation is combined with adult gastric irritation to establish a FD animal model. TNBS-induced colonic inflammation in animal pups leads to gastric hypersensitivity, delayed gastric emptying, and depression-like behaviors. Gastric irritation via iodoacetamide causes impaired gastric sensory and motor dysfunction.

*Methods.* Neonatal animals are treated with TNBS followed by treatment with 0.1% iodoacetamide for seven days at 6–8 weeks of age [21].

*Features. *This model concentrates the advantages of two kinds of methods and presents typical pathophysiological characteristics of FD, such as early satiety, delayed gastric emptying, gastric hypersensitivity, and depression-like behaviors. But it does not worsen the symptoms of FD compared to neonatal colon inflammation alone [21].

### 8.2. Neonatal Maternal Separation Followed by Adult Restraint Stress

*Rationale. *Neonatal maternal separation leads to delayed gastric emptying in adulthood. Restraint stress also suppresses antral motility and delays gastric emptying. The gastric motility may be even worse when the two models are combined together.

*Methods. *Animal pups are separated from their parents and placed in individual cages in another room for three hours daily. Restraint stress was performed for one week when the animals reached adulthood [25, 26].

*Features. *Neonatal maternal separation followed by adult restraint stress induces a more severe delay in gastric emptying compared with neonatal maternal separation or restraint stress alone. This compound model causes a relatively serious delay of gastric motility.

## 9. Which Animal Model Is Most Suited to Study FD?

**Figure 1:**
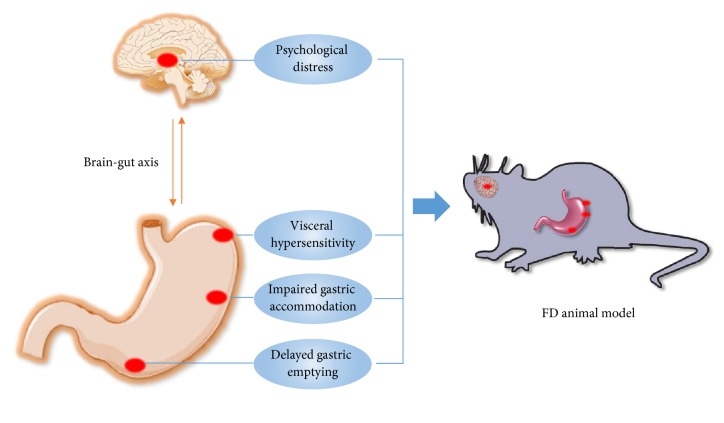
Delayed gastric emptying, impaired gastric accommodation, visceral hypersensitivity, and psychological distress are the major pathophysiologic disturbances of FD. These points are also targets of FD animal model.

An ideal animal model should mimic the disease conditions and outcomes as close as possible to the human condition. The ideal model may offer an opportunity to understand molecular mechanisms that lead to disease development and help to develop new drugs. All the above animal models of FD were established on the basis of the following features: delayed gastric emptying, impaired gastric accommodation, visceral hypersensitivity, and psychological distresses (Figure 1). Different FD animal models have different merits. For example, clonidine injection has been used mainly to induce delay in gastric emptying and BB-DP rats are used mainly for impaired gastric accommodation. Among these models, neonatal gastric irritation is the best model because it presents the main pathophysiological characteristics of FD.

It is unrealistic to expect any animal model to mimic every facet of the complex disturbances typical for FD. The decision about which particular model should be chosen to study FD will depend on the study aims and questions of interest. Compound animal model that include two or more factors that contribute to FD development may be preferable. To date few studies have used compound animal model in preclinical studies of FD. These complex models might be more promising in future studies because they can reproduce the pathogenic lesions that underlie the disease in humans from different aspects. Additionally, genetic approaches might also be used to generate animal models of FD.

## 10. Conclusion

FD adversely affects attendance and productivity in the workplace and causes enormous expense for patients and society [110]. Although a cure for this disease is still far in the future, some progress in terms of the development and characterization of FD animal models has been made. Various kinds of methods have been employed to establish FD models from different pathological perspectives. Delayed gastric emptying, impaired gastric accommodation, visceral hypersensitivity, and psychological distresses are the main targets for establishing FD models. Different animal models are better suited for modelling certain aspects of FD. The most appropriate model according to the reasons for the experiment. Although great challenges remain, the development of FD animal models that offer pathophysiological characteristics similar to FD patients should be encouraged. Good models will help us better understand the mechanisms of FD and give us a better chance to develop effective therapies.

## Figures and Tables

**Table 1:** Animal models of functional dyspepsia.

Approach	Species	Mechanism	Pharmacologic action	T/C	Year	Reference
*Drug administration animal models*						
Clonidine injection	SD rats, ICR mice, Beagle dogs	*α*2-Adrenoceptor agonists	Delays gastric emptying and intestinal transit	T	2011, 2012, 2015, 2016	[2, 6–9]
Atropine injection	SD rats, ICR mice	Muscarinic acetylcholine receptor antagonists	Delays gastrointestinal transit	T	2008, 2012	[10, 11]
Dopamine injection	ICR mice	Dopamine agonists	Delays gastric emptying	T	2012, 2016	[9, 10]
Apomorphine injection	SD rats, ICR mice	Dopamine receptor agonists	Delays gastric emptying	T	2008, 2010, 2011, 2015	[2, 11–13]
5-HT injection	ICR mice	5-HT agonists	Delays gastric emptying and reduces small intestinal motility	T	2012	[10]
5-HT_3_ receptor agonist injection	ICR mice	5-HT receptor agonists	Delays gastric emptying and reduces small intestinal motility	T	2012	[10]
CRF injection	SD rats	Stress hormone	Delays gastric emptying	T	2012	[14]
Cisplatin injection	SD rats, Swiss mice	Gastrointestinal side effects	Delays gastric emptying	T	2008, 2012	[11, 15]
Soybean oil administration	SD rats	Gastric motility	Delays gastric emptying	T	2012	[14]
*Neonatal intervention animal models*						
Neonatal gastric irritation	SD rats	Mild gastritis in early life	Delays gastric emptying, induces gastric hypersensitivity, impairs accommodation, increases depressive and anxious behaviors	C	2008, 2011, 2014, 2017,	[16–21]
Neonatal colon inflammation	SD rats	Colitis in early life	Delays gastric emptying, induces gastric hypersensitivity, increases depressive behaviors	C	2013, 2016, 2017	[21–24]
Neonatal maternal separation	Wistar rats	Childhood trauma	Delays gastric emptying	C	2015, 2016	[25, 26]
*Stress stimulation animal models*						
Restraint stress	Wistar rats, ICR mice,	Chronic stresses	Delays gastric emptying	C	2008, 2015	[2, 26–28]
Water-immersion restraint stress	SD rats, Wistar rats	Acute stresses	Delays gastric emptying	T	2013, 2016	[25, 29]
Tail clamping	SD rats, Wistar rats	Chronic stresses	Delays gastric emptying, increases anxious behaviors	C	2011, 2016	[30–32]
CMUS	SD rats	Chronic stresses	Increases depressive behaviors	C	2017	[33]
*Other intervention animal models*						
Gastric distension	Wistar rats	Abdominal muscle	Induces visceral hyperalgesia	C	2011	[34]
Laparotomy	SD rats	Local inflammation	Delays gastrointestinal transit	C	2008, 2015	[11, 35]
Duodenal acidification	SD rats	Duodenum	Induces gastric hypersensitivity	T	2014	[36]
Chronic high-fat feeding	SD rats	Gastric motility	Delays gastric emptying	C	2015	[37]
*Special species animal models*						
FSL rats	FSL rats	Genetic depression	Delays gastric emptying, impairs accommodation, increases depressive behaviors	C	2005	[38]
WKY rats	WKY rats	Genetic anxiety	Impairs accommodation, increases anxious behaviors	C	2007	[39]
BB-DP rats	BB-DP rats	Genetic impaired accommodation	Impairs gastric accommodation	C	2014	[40]
*Compound factors animal models*						
Neonatal colon inflammation followed by adult gastric irritation	SD rats	Colitis in early life and gastritis	Delays gastric emptying, induces gastric hypersensitivity, increases depressive behaviors	C	2017	[21]
Neonatal maternal separation followed by adult restraint stress	Wistar rats	Childhood trauma and chronic stresses	Delays gastric emptying	C	2015, 2016	[25, 26]

T, temporary; C, continuous; SD, Sprague-Dawley; ICR, Institute of Cancer Research; 5-HT, 5-Hydroxytryptamine; CRF, corticotropin-releasing factor; CMUS, chronic mild unpredictable stimulation; FSL, Flinders Sensitive Line; WKY, Wistar Kyoto; BB-DP, BioBreeding diabetes-prone.

## Abbreviations

FD: Functional dyspepsia
5-HT: 5-Hydroxytryptamine
m-CPBG: m-Chlorophenylbiguanide
CRF: Corticotropin-releasing factor
TNBS: Trinitrobenzene sulfonic acid
HPA: Hypothalamus-pituitary-adrenal
ACTH: Adrenocorticotrophic hormone
CMUS: Chronic mild unpredictable stimulation
HCI: Hydrochloric acid
TGR5: Bile acid receptor 1
nNOS: Neuronal nitric oxide synthase
FSL: Flinders Sensitive Line
WKY: Wistar Kyoto
BB-DP: BioBreeding diabetes-prone.
